# Nutritional status, body composition and bone health in treatment-naïve transgender and gender-diverse adolescents

**DOI:** 10.3389/fnut.2026.1937542

**Published:** 2026-09-23

**Authors:** Yael Issan, Ophir Borger, Liat Perl, Asaf Oren, Anat Segev-Becker, Dor Shoshan, Yaffa Elbaum-Shiff, Tair Ben-Porat, Yael Lebenthal

**Affiliations:** 1 The Institute of Pediatric Endocrinology, Diabetes and Metabolism, Dana-Dwek Children’s Hospital, Tel Aviv Sourasky University Medical Center, Tel Aviv, Israel; 2 The Nutrition and Dietetics Unit, Tel Aviv Sourasky University Medical Center, Tel Aviv, Israel; 3 Faculty of Social Welfare and Health Sciences, School of Public Health, University of Haifa, Haifa, Israel; 4 Gray Faculty of Medical and Health Sciences, The School of Medicine, Tel Aviv University, Tel Aviv, Israel

**Keywords:** bone mineral density (BMD), calcium intake, gender dysphoria (GD), gonadotropin-releasing hormone analogue (GnRHa), nutritional status, transgender adolescents, treatment-naïve, vitamin D

## Abstract

**Background:**

Gonadotropin-releasing hormone analogue (GnRHa)-induced gonadal suppression is an established component of gender-affirming care for transgender and gender-diverse (TGD) adolescents. Impaired bone health has been documented in treatment-naïve TGD adolescents prior to GnRHa intervention, yet its nutritional determinants remain poorly characterized.

**Objective:**

To characterize nutritional status, body composition, and bone mineral density (BMD) in treatment-naïve TGD adolescents prior to GnRHa initiation, and to identify predictors of lumbar spine BMD z-scores.

**Methods:**

This retrospective cross-sectional study included 147 treatment-naïve TGD adolescents (59 AMAB[TGD-Female] and 88 AFAB[TGD-Male]) attending a national pediatric gender clinic. Assessments included anthropometrics, body composition, 24-h dietary calcium intake (compared to the age-specific dietary reference intake [DRI]), micronutrient status, physical activity, sleep duration, and lumbar spine BMD z-scores by dual-energy X-ray absorptiometry. Multiple linear regression identified predictors of BMD z-score, with sex assigned at birth included as a covariate. Descriptive analyses were conducted by sex assigned at birth.

**Results:**

The median age was 15.5 years (IQR, 14.1–16.7), and 83.7% were fully pubertal. The median daily calcium intake was 400 mg/d (31% of the DRI), with 99.3% consuming below the DRI. Vitamin D deficiency/insufficiency was present in 91.5% of participants. Of the 102 participants with complete micronutrient data, 63.7% had ≥ 2 nutritional deficiencies. Body composition assessment revealed a below-average muscle-to-fat ratio in both groups (median z-score −0.74) and a higher fat mass percentage in AFAB compared to AMAB adolescents (28.6% vs. 17.4%; *p* < 0.001). The overall median BMD z-score was −0.70 (IQR, −1.30 to 0.10), with the AMAB adolescents having lower BMD z-scores than the AFAB adolescents (−1.00 vs. −0.40; *p* = 0.001), and 55.8% of the former having below-average/low BMD values compared with 33.0% of the latter (*p* = 0.015). The multivariable regression revealed that the BMI z-score (*B* = 0.403; *p* < 0.001) and sex assigned at birth (*B* = 0.372; *p* = 0.019) were the only independent predictors of the BMD z-score (R^2^ = 0.344, adjusted R^2^ = 0.318).

**Conclusion:**

Treatment-naïve TGD adolescents exhibit nutritional deficiencies, unfavorable body composition, and reduced BMD prior to GnRHa initiation, particularly those assigned male at birth. BMI z-scores and sex assigned at birth independently predicted BMD, highlighting the need for targeted pre-treatment nutritional and lifestyle interventions.

## Introduction

Gonadal hormone suppression with gonadotropin-releasing hormone analogues (GnRHa) is an established component of gender-affirming care for adolescents with gender dysphoria, yet its administration during a developmental window in which 25–50% of peak bone mass is accrued raises fundamental concerns about long-term skeletal health that remain incompletely understood (1–4). Several cross-sectional studies have demonstrated that transgender and gender diverse (TGD) adolescents exhibit a higher prevalence of low bone mineral density (BMD) for age compared with cisgender peers, even before initiating any medical intervention (5–7). This pre-existing skeletal vulnerability is most pronounced among those assigned male at birth (AMAB; TGD-Female), in whom BMD z-scores are frequently below the population mean prior to gonadal hormone suppression (6, 7). TGD adolescents assigned female at birth (AFAB; TGD-Male) tend to have BMD closer to age-matched normative data, although a subset of that group was reported to also demonstrate suboptimal bone mass (8, 9). Importantly, GnRHa therapy can further exacerbate this trajectory, since treatment is consistently associated with declines in BMD z-scores and reduced bone mass accrual relative to age-matched cisgender peers who continue pubertal progression (8, 10, 11). However, the long-term implications for peak bone mass attainment and adult fracture risk remain uncertain (8, 10, 11).

The etiology of impaired pre-treatment skeletal status in TGD adolescents is likely multifactorial. Nutritional and lifestyle factors, including inadequate dietary calcium intake, vitamin D deficiency or insufficiency, reduced weight-bearing physical activity, poor sleep, lower BMI, and unfavorable body composition, have been proposed as contributors to compromised bone health in this population (6, 7, 9, 11, 12). Calcium and vitamin D are critical substrates for bone mineralization in adolescents, yet most of them fail to meet the recommended dietary intake of 1300 mg/d and 600 IU/d, respectively (13–15). Unhealthy dietary patterns, food insecurity, disordered eating behaviors, and psychosocial stressors such as minority stress and weight-based bullying may further exacerbate nutritional deficits and physical inactivity among TGD adolescents (6, 16–18). In addition, impaired body composition, characterized by reduced lean mass and a lower muscle-to-fat ratio (MFR), has been consistently reported across TGD cohorts and shown to be independently associated with lower BMD (6, 7, 9).

Despite the growing awareness of these vulnerabilities, evidence for characterizing the pre-treatment skeletal and nutritional status of TGD adolescents remains limited. Existing studies are predominantly small, single-center case series or cross-sectional analyses that lack detailed nutritional assessment, body composition data, and evaluation of multiple lifestyle determinants of bone health within the same cohort (6, 7, 9). In particular, no study has assessed dietary calcium intake using validated nutritional software in combination with dual-energy X-ray absorptiometry (DXA)-derived BMD, bioelectrical impedance analysis (BIA)-derived body composition, and micronutrient status in a treatment-naïve TGD cohort. Consequently, the relative contributions of nutritional, behavioral, and anthropometric factors to skeletal health before treatment remain poorly defined. Therefore, the aim of this study was to characterize nutritional status, body composition, and bone health in a large cohort of treatment-naïve TGD adolescents prior to GnRHa initiation and to identify independent predictors of the lumbar spine BMD z-score.

## Materials and methods

### Study design and population

This retrospective cross-sectional study was based on electronic medical record review of treatment-naïve TGD adolescents who attended the Israeli Children and Adolescents Gender Clinic, a national referral center for TGD youth, between January 2023 and December 2025. Our clinic provides standardized multidisciplinary care and, as part of the baseline evaluation prior to initiation of medical therapy, treatment-naïve TGD adolescents undergo anthropometric assessment, medical and psychosocial evaluations, dietary consultation, body composition analysis by BIA, and DXA assessment before initiation of GnRHa therapy. Medical records were reviewed to identify those meeting all inclusion criteria.

### Ethics

The study protocol was approved by the Tel Aviv Sourasky Medical Center Ethics Committee (TLV-0293-17), which waived the requirement for informed consent given the retrospective nature of the study. All procedures were conducted in accordance with the principles of Good Clinical Practice and the Declaration of Helsinki.

### Inclusion and exclusion criteria

Individuals were included if they were treatment-naïve at the time of clinic attendance, defined as no prior exposure to GnRHa, no gender-affirming hormones, nor any other hormonal intervention, and aged between 10 and 18 years. Inclusion additionally required a completed nutritional assessment, with dietary calcium intake calculation conducted by a registered dietitian, laboratory biomarkers of nutritional status on record, and an available DXA report of the lumbar spine. Of the total of 311 records that were reviewed, 116 individuals attended only once (36 AMAB, 80 AFAB) and were excluded. Of the remaining 195 adolescents, a further 48 were excluded due to unavailable DXA and/or incomplete nutritional assessment (25 AMAB, 23 AFAB). These exclusions left a total of 147 individuals who met all criteria and were included in the analysis, of whom 59 had been assigned male at birth (AMAB; TGD-Female) and 88 were assigned female at birth (AFAB; TGD-Male). Descriptive analyses were conducted by sex assigned at birth.

### Anthropometric and body composition parameters

Anthropometric and body composition data were extracted from clinical measurements recorded at the time of clinic attendance. Height z-scores were calculated using the 2000 CDC growth charts and are reported in Table 1. No participant had a height z-score below −2.0, and formal height-age BMD adjustment per ISCD 2019 guidance was not indicated. Body mass index (BMI) was calculated as weight in kilograms divided by height in meters squared (kg/m^2^) and converted to sex-assigned-at-birth and age-specific z-scores by means of the 2000 CDC growth charts (19). Weight status was classified as underweight (BMI z-score ≤ −1.65), healthy weight (−1.65 to 1.04), overweight (1.04 to 1.65), or obese ( ≥ 1.65) (20). Body composition was assessed with the Tanita MC-780MA multi-frequency BIA device (Tanita Corporation, Tokyo, Japan), which provides whole-body and segmental fat and muscle analysis. Appendicular skeletal muscle mass (ASMM) was calculated as the sum of muscle mass across the four limbs, and the muscle-to-fat ratio (MFR) was derived as ASMM (kg) divided by total fat mass (kg) and converted to sex-assigned-at-birth and age-specific z-scores by means of the McCarthy normative reference data (21). BIA data were available in 100 of 147 records (68.0%); the remaining 47 were irrecoverably lost due to a hardware failure during the study period.

**TABLE 1:** Demographic and anthropometric characteristics of treatment-naïve TGD adolescents stratified by sex assigned at birth.

Characteristic	Total (*n* = 147)	TGD-female (AMAB, *n* = 59)	TGD-male (AFAB, *n* = 88)	*P*-value
Age at assessment, years	15.5 (14.1–16.7)	15.9 (14.4–16.9)	15.2 (13.9–16.2)	0.051
Height z-score	0.13 (−0.68 to 0.79)	−0.13 (−0.96 to 0.74)	0.28 (−0.58 to 0.79)	0.287
BMI z-score	0.27 (−0.68 to 1.10)	−0.34 (−0.78 to 0.88)	0.46 (−0.46 to 1.42)	**0.025**
Weight status, n (%)				
Underweight (z ≤ −1.65)	12 (8.2)	7 (11.9)	5 (5.7)	0.374
Healthy weight (−1.65 < z < 1.04)	96 (65.3)	41 (69.5)	55 (62.5)	
Overweight (1.04 ≤ z < 1.65)	14 (9.5)	4 (6.8)	10 (11.4)	
Obese or severely obese (z ≥ 1.65)	25 (17.0)	7 (11.9)	18 (20.5)	
Psychiatric conditions, *n* (%)	84 (57.1)	36 (61.0)	48 (54.5)	0.544
Pubertal stage, n (%)				
Tanner 1 (pre-pubertal)	3 (2.0)	1 (1.7)	2 (2.3)	0.459
Tanner 2–4 (in puberty)	21 (14.3)	11 (18.6)	10 (11.4)	
Tanner 5 (fully pubertal)	123 (83.7)	47 (79.7)	76 (86.4)	
Socioeconomic position, n (%)				
Low (cluster 1–4)	5 (3.4)	3 (5.1)	2 (2.3)	0.606
Medium (cluster 5–7)	53 (36.1)	22 (37.3)	31 (35.2)	
High (cluster 8–10)	89 (60.5)	34 (57.6)	55 (62.5)	

Data are presented as median (interquartile range) for continuous variables and *n* (%) for categorical variables. Continuous variables were compared by the Mann-Whitney U test, and categorical variables by the chi-square test. A *p*-value of < 0.05 was considered significant. Bold indicates significant. Weight status was defined per 2000 CDC BMI-for-sex and age growth charts (19), and socioeconomic position cluster per Israel Central Bureau of Statistics (range 1–10) (28). AFAB, assigned female at birth; AMAB, assigned male at birth; IQR, interquartile range; TGD, transgender and gender diverse.

### Nutritional intake and micronutrient status

Nutritional assessment was conducted as part of a structured multidisciplinary evaluation during the first clinic attendance. The dietary assessment was performed by a registered dietitian (Y.I. and O.B.) and comprised a comprehensive consultation that included a structured face-to-face a 24-h dietary recall of approximately 1 h, during which patients were asked to describe their typical dietary intake. Calcium intake was calculated in milligrams per day by means of Tzameret version 6.0 software (Israel Ministry of Health, Jerusalem, Israel) (22), a validated national nutritional database. All assessments were conducted on clinic weekdays; weekend dietary patterns were not captured. Although a single 24-h dietary recall does not provide the same estimate of habitual intake as repeated dietary records, the structured interviewer-administered format allowed standardized assessment of reported dietary intake in this cohort. Daily calcium intake was compared to the age-specific dietary reference intake (DRI) of 1300 mg/d for adolescents aged 9 to 18 years (13, 14). Given the uniformly low reported calcium intake observed across the cohort, intake was categorized by percentage of the DRI in order to examine dose-response relationships: < 25% DRI ( < 325 mg/d), 25–50% DRI (325–650 mg/d), and > 50% DRI ( > 650 mg/d). Laboratory values were extracted from fasting blood samples drawn as part of the routine pre-treatment clinical evaluation and documented in the medical record. The following nutritional biomarkers were extracted: serum 25-hydroxyvitamin D (calcifediol, nmol/L), serum ferritin (ng/mL), cobalamin (vitamin B12, pg/mL), and folic acid (vitamin B9, ng/mL). Vitamin D status was classified as deficient ( < 50 nmol/L), insufficient (50–75 nmol/L), or sufficient ( ≥ 75 nmol/L) (23). Iron deficiency was defined as serum ferritin < 15 ng/mL (24). Vitamin B12 was classified as deficient ( < 200 pg/mL) or borderline (200–300 pg/mL) and sufficient ( > 300 pg/mL) (25). Folic acid was classified as deficient ( < 3 ng/mL), borderline (3 to < 6 ng/mL) or sufficient ( ≥ 6 ng/mL) (26).

### Bone health assessment

Lumbar spine (L1–L4) BMD was assessed by DXA using a GE Lunar Prodigy Primo densitometer (GE Healthcare, Madison, WI, USA) at external imaging centers as part of the standard pre-treatment clinical workup. All BMD z-scores were derived from GE Lunar Prodigy Primo manufacturer-specific pediatric normative references, ensuring consistency of measurements across all participants. BMD values (g/cm^2^) were expressed as sex-assigned-at-birth and age-specific z-scores using manufacturer pediatric normative references and categorized per ISCD 2019 Pediatric Official Positions as normal (z > −1.0), below average (−2.0 ≤ z < −1.0), or low BMD for chronological age (z ≤ −2.0) (27). The dietary consultation and laboratory assessment were conducted prior to and in direct temporal proximity to the DXA evaluation, enabling integrated characterization of nutritional status alongside skeletal health at the time of the first clinic attendance before initiation of GnRHa therapy.

### Sociodemographic, clinical, and lifestyle characteristics

Sociodemographic, clinical, and lifestyle data were extracted from electronic medical records. Sociodemographic variables included age and sex assigned at birth, as well as socioeconomic position (SEP), which was derived from each participant’s residential area according to the Israel Central Bureau of Statistics socioeconomic index (range 1–10) and grouped into low (cluster 1–4), medium (cluster 5–7), and high (cluster 8–10) tertiles (28). Clinical data included pubertal stage (Tanner stage) and relevant medical history documented at the initial clinic visit. Psychiatric conditions assessed in the medical records included depression, anxiety, obsessive-compulsive disorder (OCD), history of suicidal ideation or suicide attempt, and self-harm. Participants were classified as having a psychiatric condition if at least one of these conditions was documented. Lifestyle behavior data, including physical activity and sleep duration, were extracted from self-reported information documented in the medical record. Physical activity was classified as meeting or not meeting World Health Organization (WHO) guidelines for adolescents, defined as ≥ 60 min per day of moderate-to-vigorous intensity aerobic physical activity on most days of the week (29). Sleep duration was classified as adequate or inadequate relative to age-appropriate pediatric recommendations (9–11 h per night for ages 6–12 years and 8–10 h per night for ages 13–18 years) (30).

### Statistical analysis

All analyses were performed with SPSS version 29.0.1 (IBM Corp, Armonk, NY, USA). Continuous variables were assessed for normality by the Shapiro–Wilk test and are reported as median (interquartile range [IQR]). Categorical variables are reported as frequency and percentage. Non-parametric tests were used since several key variables were not normally distributed. Between-group differences (AMAB vs AFAB) were assessed by the Mann–Whitney U test for continuous variables and the chi-square test for categorical variables. Fisher’s exact test was applied where expected cell counts were below 5. Associations between candidate predictors and lumbar spine BMD z-score were evaluated by the Spearman rank correlation (ρ). Differences in BMD z-score across calcium intake strata were assessed by the Kruskal–Wallis test, together with *post hoc* pairwise Mann–Whitney U tests where appropriate.

Independent predictors of lumbar spine BMD z-score were examined by multiple linear regression. Covariates were selected *a priori* based upon established determinants of bone health in adolescents and study hypotheses, including BMI z-score, MFR z-score, daily calcium intake, serum vitamin D, age, and sex assigned at birth. Tanner stage was not included given that 83.7% of participants were Tanner stage 5 (insufficient variance for reliable estimation), the small number of Tanner 1–4 participants (*n* = 24), and high collinearity with age (Spearman ρ = 0.52, *p* < 0.001). Two models were constructed: Model 1 included all covariates (*n* = 90 complete cases), while Model 2 excluded the MFR z-score in order to maximize sample size (*n* = 132) due to missing BIA data (32.0%); Model 2 is presented as the primary model. Model performance was evaluated by F statistics, R^2^, and adjusted R^2^. Regression assumptions were assessed, including normality of residuals (Omnibus test), absence of autocorrelation (Durbin–Watson statistic), homoscedasticity, and absence of multicollinearity (variance inflation factor). Influential observations were assessed using Cook’s distance. A sensitivity analysis restricted to Tanner stage 5 participants was conducted to assess whether findings were driven by differences in pubertal maturation. A sensitivity analysis testing the BMI z-score × sex interaction term was conducted to assess the appropriateness of the pooled modelling approach. All tests were two-tailed, with statistical significance defined as *p* < 0.05.

## Results

The overall median age of the cohort was 15.5 years (IQR, 14.1–16.7). The AMAB adolescents were slightly older than the AFAB adolescents (15.9 vs. 15.2 years), but the difference did not reach a level of significance (*p* = 0.051). The majority of the individuals were fully pubertal (Tanner stage 5, *n* = 123, 83.7%), and 84 (57.1%) had at least one psychiatric condition. Most of the individuals came from a high socioeconomic position (*n* = 89, 60.5% SEP cluster 8–10). The median BMI z-score was significantly lower in the AMAB group than in the AFAB group (−0.34 vs. 0.46; *p* = 0.025). Weight status distribution did not differ significantly between groups, nor were any between-group differences found in pubertal stage, psychiatric conditions, or SEP. The majority of the individuals (*n* = 105, 71.4%) did not meet WHO physical activity guidelines, and 58 (39.5%) reported inadequate sleep duration, neither parameter differing significantly between groups. Patient characteristics stratified by sex assigned at birth are presented in Table 1. The overall median MFR z-score was −0.74 (IQR, −1.38 to −0.06), with no significant between-group difference. Fat mass percentage was significantly higher in the TGD-Male (AFAB) group than in the TGD-Female (AMAB) group (median 28.6% vs. 17.4%; *p* < 0.001). Body composition data are presented in Table 2.

**TABLE 2:** Body composition, nutritional intake, micronutrient status, bone health, and lifestyle behaviors in treatment-naïve TGD adolescents stratified by sex assigned at birth.

Variable	Total	TGD-female (AMAB, *n* = 59)	TGD-male (AFAB, *n* = 88)	*P-*value
Body composition (BIA)a				
MFR z-score	−0.74 (−1.38 to −0.06)	−0.59 (−1.21 to 0.31)	−0.84 (−1.42 to −0.23)	0.125
Fat mass, %	25.8 (19.4–32.2)	17.4 (16.0–22.0)	28.6 (24.6–37.1)	**< 0.001**
Nutritional intake and micronutrients				
Calcium intake, mg/d	400 (256–557)	400 (245–557)	400 (292–540)	0.627
< 25% DRI, < 325 mg/d, *n* (%)	56 (38.1)	25 (42.4)	31 (35.2)	0.575
25–50% DRI, 325–650 mg/d, *n* (%)	70 (47.6)	25 (42.4)	45 (51.1)	
> 50% DRI, > 650 mg/d, *n* (%)	21 (14.3)	9 (15.3)	12 (13.6)	
Vitamin D, nmol/L^b^	50.6 (35.0–60.1)	52.2 (38.7–62.3)	50.0 (35.0–59.8)	0.551
Deficient, < 50 nmol/L, *n* (%)	68 (47.9)	27 (46.6)	41 (48.8)	0.414
Insufficient, 50–75 nmol/L, *n* (%)	62 (43.7)	28 (48.3)	34 (40.5)	
Sufficient, ≥ 75 nmol/L, *n* (%)	12 (8.5)	3 (5.2)	9 (10.7)	
Serum ferritin, ng/mL^c^	27.3 (14.6–41.5)	39.0 (23.2–58.5)	22.8 (10.9–32.6)	**< 0.001**
Iron deficient, *n* (%)	32 (25.4)	5 (10.4)	27 (34.6)	**0.003**
Vitamin B12, pg/mL^d^	340 (280–426)	312 (269–403)	360 (288–431)	0.294
Deficient, < 200 pg/mL, *n* (%)	5 (3.8)	2 (4.0)	3 (3.7)	0.566
Borderline, 200–300 pg/mL, *n* (%)	43 (32.6)	19 (38.0)	24 (29.3)	
Sufficient, > 300 pg/mL, *n* (%)	84 (63.6)	29 (58.0)	55 (67.1)	
Folic acid, ng/mL^e^	7.8 (5.4–10.0)	8.0 (5.6–11.1)	7.8 (5.2–10.0)	0.443
Deficient, < 3 ng/mL, *n* (%)	7 (5.8)	3 (6.4)	4 (5.4)	0.972
Borderline, 3–6 ng/mL, *n* (%)	29 (24.0)	11 (23.4)	18 (24.3)	
Sufficient, ≥ 6 ng/mL, *n* (%)	85 (70.2)	33 (70.2)	52 (70.3)	
Bone health				
BMD z-score, L1–L4	−0.70 (−1.30 to 0.10)	−1.00 (−1.40 to −0.40)	−0.40 (−1.20 to 0.60)	**0.001**
Normal, BMD z-score > −1.0, *n* (%)	85 (57.8)	26 (44.0)	59 (67.0)	**0.015**
Below average (−2.0 ≤ BMD z-score < −1.0), *n* (%)	53 (36.0)	27 (45.7)	26 (29.6)	
Low BMD for age (z ≤ −2.0), *n* (%)	9 (6.1)	6 (10.1)	3 (3.4)	
Lifestyle behaviors				
Not meeting PA guidelines, *n* (%)	105 (71.4)	45 (76.3)	60 (68.2)	0.380
Inadequate sleep duration, *n* (%)	58 (39.5)	25 (42.4)	33 (37.5)	0.674

Data are presented as median (IQR) for continuous variables and *n* (%) for categorical variables. Continuous variables were compared by the Mann-Whitney U test, and categorical variables by the chi-square test; Fisher’s exact test was applied where expected cell counts were below 5. BMD z-scores were categorized per ISCD 2019 Pediatric Official Positions: normal (z > −1.0), below average bone density (−2.0 ≤ z < −1.0), and low BMD for chronological age (z ≤ −2.0); “below average bone density” is an ISCD-defined descriptive category and does not constitute a clinical diagnosis.

^a^BIA data was available for *n* = 100 (36 AMAB, 64 AFAB).

^b^Vitamin D data were available for *n* = 142 (58 AMAB, 84 AFAB).

^c^Serum ferritin data were available for *n* = 126 (48 AMAB, 78 AFAB).

^d^Vitamin B12 data were available for *n* = 132 (50 AMAB, 82 AFAB).

^e^Folic acid data were available for *n* = 121 (47 AMAB, 74 AFAB). AFAB, assigned female at birth; AMAB, assigned male at birth; BIA, bioelectrical impedance analysis; BMD, bone mineral density; DRI, dietary reference intakes; IQR, interquartile range; MFR, muscle-to-fat ratio; PA, physical activity. A *p-*value of < 0.05 was considered significant. Bold indicates significant.

Of the 116 individuals excluded for attending only once, the excluded group did not differ significantly from included participants on age (median 15.4 vs. 15.5 years; *p* = 0.347) or socioeconomic position (*p* = 0.585), though the excluded group had a significantly higher proportion of AFAB adolescents (69.0% vs. 59.9%; *p* = 0.021). Within the included cohort, participants with versus without BIA data did not differ significantly on age, BMI z-score, sex distribution, or BMD z-score (all *p* > 0.14). Participants included versus excluded from the primary regression model (*n* = 132 vs. *n* = 15) did not differ significantly on age, BMI z-score, or BMD z-score (all *p* > 0.46), supporting the validity of the complete-case approach.

### Nutritional intake and micronutrient status

The median daily calcium intake was 400 mg/d (IQR, 256–557 mg/d), corresponding to 31% of the age-specific DRI of 1300 mg/d, with no significant difference between the groups (Figure 1 and Table 2). When categorized by intake strata, 56 (38.1%) individuals consumed fewer than 25% of the DRI ( < 325 mg/d), 70 (47.6%) consumed 25–50% of the DRI (325–650 mg/d), and 21 (14.3%) consumed more than 50% of the DRI ( > 650 mg/d), with no significant difference in distribution between groups. For descriptive comparison, the reported median calcium intake in this cohort was less than half the median reported for the general Israeli adolescent population in the MABAT Youth 2015–2016 national survey (400 vs. 899 mg/d, respectively). The proportion meeting the DRI was also markedly lower than in the general Israeli adolescent population (0.7% vs. 24.5%). The median serum vitamin D level was 50.6 nmol/L (IQR, 35.0–60.1). Vitamin D status was deficient ( < 50 nmol/L) in 68 (47.9%) individuals, insufficient (50–75 nmol/L) in 62 (43.7%), and sufficient ( ≥ 75 nmol/L) in only 12 (8.5%), with no significant difference in distribution between groups. Serum ferritin was significantly lower in the AFAB group than in the AMAB group (median 22.8 vs. 39.0 ng/mL; *p* < 0.001), and iron deficiency (ferritin < 15 ng/mL) was present in 27 (34.6%) of the AFAB group compared with 5 (10.4%) of the AMAB group (*p* = 0.003). A total of 48 (36.4%) of the cohort had deficient or borderline vitamin B12 levels and 36 (29.8%) had deficient or borderline folic acid levels, with no significant between-group differences. Among the 102 individuals with complete data for all four nutritional biomarkers, 65 (63.7%) had two or more co-occurring deficiencies or borderline values. Only four participants (3.9%) had no nutritional deficiency across any marker, whereas 33 (32.4%) had one, 40 (39.2%) had two, 23 (22.5%) had three, and 2 (2.0%) met deficiency or borderline thresholds for all four biomarkers simultaneously. The nutritional profile of this cohort is presented in Table 2.

**FIGURE 1:**
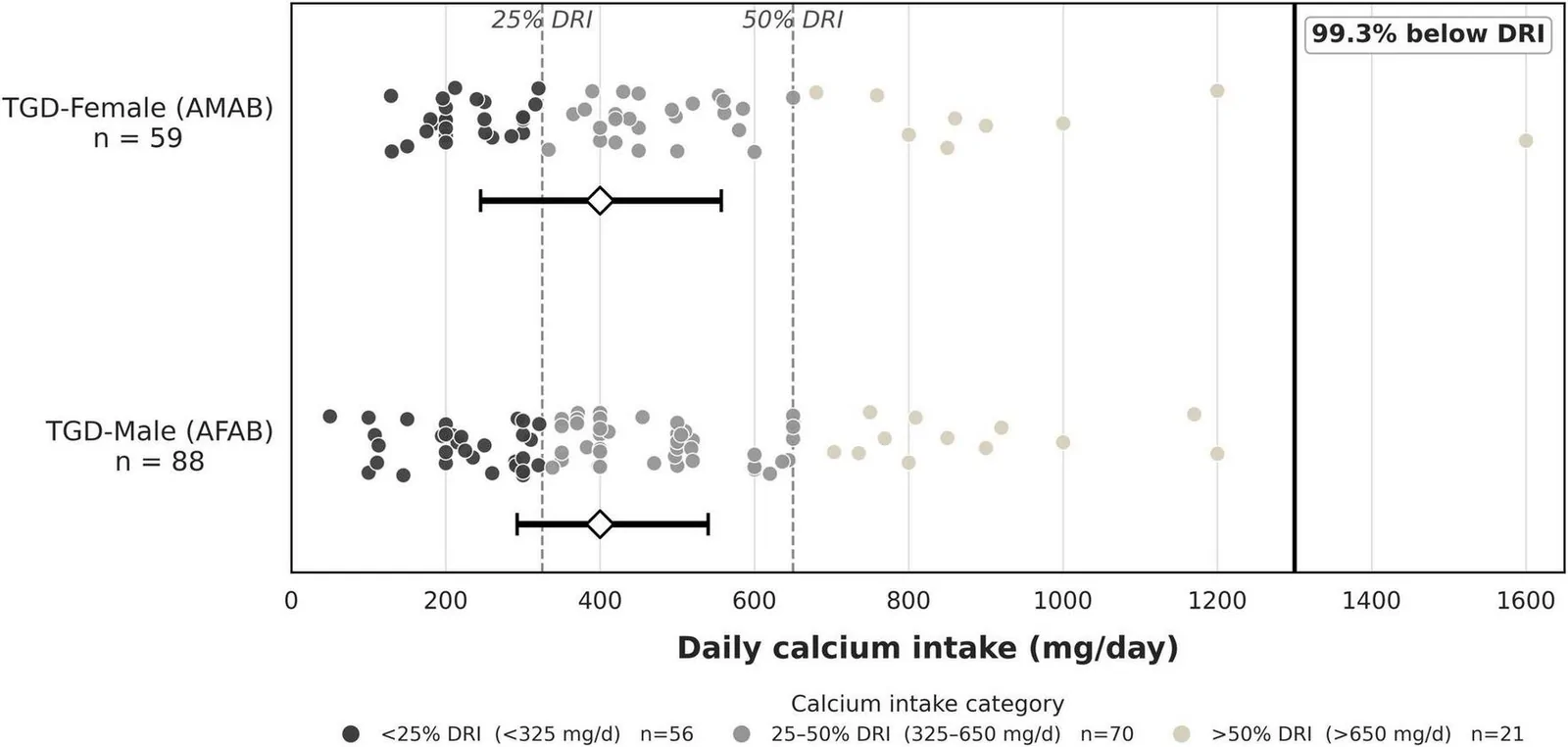
Daily calcium intake relative to dietary reference intake among treatment-naïve transgender and gender-diverse adolescents. AMAB, assigned male at birth; AFAB, assigned female at birth; DRI, dietary reference intake; TGD, transgender and gender-diverse.

### Bone mineral density

The overall median BMD z-score was −0.70 (IQR, −1.30 to 0.10). The AMAB group had lower median BMD z-scores than the AFAB group (−1.00 [IQR, −1.40 to −0.40] vs. −0.40 [−1.20 to 0.60], respectively; *p* = 0.001). The BMD category distribution differed significantly between groups (*p* = 0.015): 33 (55.8%) of the AMAB group had below-average or low BMD (45.7% below average, 10.1% low), compared with 29 (33.0%) of the AFAB group (29.6% below average, 3.4% low). The distribution of BMD z-scores differed markedly between groups, with the former showing a leftward shift toward lower values compared to the latter (Figure 2). The interquartile range of the AMAB group (−1.40 to −0.40) fell almost entirely within the below-average BMD zone, whereas that of the AFAB group (−1.20 to 0.60) spanned from below-average into the normal range. Bivariate analyses revealed significant positive associations of the BMD z-score with the BMI z-score (ρ = 0.551; *p* < 0.001), and fat mass (ρ = 0.403; *p* < 0.001), and a significant inverse correlation with the MFR z-score (ρ = −0.269; *p* = 0.010). The overall bone health data are presented in Table 2.

**FIGURE 2:**
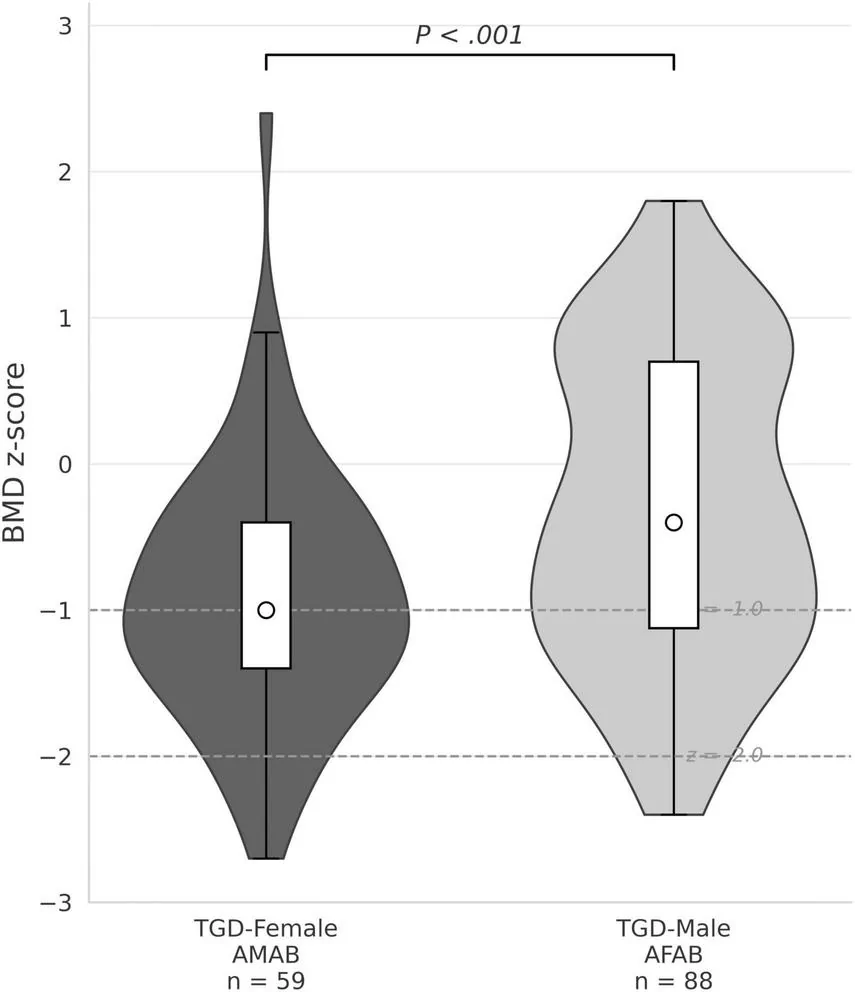
Distribution of lumbar spine bone mineral density (BMD) z-scores in treatment-naïve transgender and gender-diverse adolescents stratified by sex assigned at birth. Violin plots display the kernel density estimate of the BMD z-score distribution for TGD-Female [assigned male at birth (AMAB)] and TGD-Male [assigned female at birth (AFAB)]. Boxes span the interquartile range (IQR); whiskers extend to 1.5 × IQR; white circles indicate group medians. Horizontal dashed lines indicate International Society for Clinical Densitometry (ISCD) 2019 Pediatric Thresholds: below average bone density (*z* = −1.0) and low BMD for chronological age (*z* = −2.0). Between-group comparison by Mann-Whitney U test: *p* = 0.001. AMAB, assigned male at birth; AFAB, assigned female at birth; BMD, bone mineral density.

### Predictors of BMD z-score

In Model 1 (*n* = 90), the BMI z-score (*B* = 0.479, SE = 0.134; *p* < 0.001) and sex assigned at birth (*B* = 0.453, SE = 0.188; *p* = 0.018) were significant independent predictors of the BMD z-score, whereas MFR z-score was not (*B* = 0.247, SE = 0.179; *p* = 0.170) (R^2^ = 0.288, adjusted R^2^ = 0.237; F_6,83_ = 5.60; *p* < 0.001). In the primary model (Model 2; *n* = 132), which excluded the MFR z-score, both the BMI z-score (*B* = 0.403, SE = 0.063; *p* < 0.001) and sex assigned at birth (*B* = 0.372, SE = 0.156; *p* = 0.019) remained the only significant independent predictors (R^2^ = 0.344, adjusted R^2^ = 0.318; F_5,126_ = 13.24; *p* < 0.001). The BMD z-score increased by 0.40 for each one-unit increase in the BMI z-score after adjustment in Model 2. Being AFAB was independently associated with a higher BMD z-score in both models. Age, vitamin D level, and calcium intake were not significant independent predictors in either model. Regression results are presented in Table 3. In a sensitivity analysis restricted to Tanner stage 5 participants (*n* = 111), BMI z-score remained a significant independent predictor (*B* = 0.370; *p* < 0.001); sex assigned at birth was no longer statistically significant (*B* = 0.203; *p* = 0.243), likely reflecting reduced statistical power, as the point estimate remained in the same direction and of similar magnitude.

**TABLE 3:** Multivariable linear regression of independent predictors of lumbar spine BMD z-scores.

Predictor	Model 1 (*n* = 90)				Model 2 (*n* = 132)			
	*B*	SE	*p*	95% CI	*B*	SE	*P*	95% CI
BMI z-score	**0.479**	0.134	**< 0.001**	0.212 to 0.745	**0.403**	0.063	**< 0.001**	0.280 to 0.527
Sex assigned at birth (AMAB)^a^	**0.453**	0.188	**0.018**	0.078 to 0.827	**0.372**	0.156	**0.019**	0.063 to 0.681
MFR z-score^b^	0.247	0.179	0.170	−0.108 to 0.603	–	–	–	–
Age, years	0.006	0.046	0.888	−0.084 to 0.097	−0.048	0.036	0.189	−0.120 to.024
Vitamin D, nmol/L	0.005	0.006	0.417	−0.007 to 0.017	0.004	0.005	0.338	−0.005 to.013
Calcium intake, mg/d	−0.000	0.000	0.839	−0.001 to 0.001	0.000	0.000	0.792	−0.000 to.001
Model statistics	R^2^ = 0.288; aR^2^ = 0.237; *F* (6,83) = 5.60; *p* < 0.001				R^2^ = 0.344; aR^2^ = 0.318; *F* (5,126) = 13.24; *p* < 0.001			

^a^Sex assigned at birth is coded AMAB = 1, AFAB = 2; B = unstandardized regression coefficient; a positive B indicates a higher BMD z-score associated with being AFAB (coded 2); reference category is AMAB (coded 1).

^b^MFR z-score was included solely in Model 1 and not in Model 2 due to missing BIA data (32.0%). Durbin-Watson: Model 1 = 1.96; Model 2 = 1.99. Residuals were normally distributed in both models (Omnibus (*p* > 0.05). AFAB, assigned female at birth; AMAB, assigned male at birth; adj, adjusted; BMD, bone mineral density; BMI, body mass index; CI, confidence interval; MFR, muscle-to-fat ratio; SE, standard error. Significant predictors (*p* < 0.05) are shown in bold.

## Discussion

This cross-sectional study aimed to characterize nutritional status, body composition, and bone mineral density (BMD) in 147 treatment-naïve TGD adolescents prior to GnRHa initiation. We also sought to identify predictors of lumbar spine BMD z-scores. The findings revealed that pre-existing nutritional and skeletal vulnerabilities were prevalent and largely uniform across the cohort. Reported calcium intake was substantially below recommendations, fewer than one in ten participants had sufficient levels of vitamin D, and the majority had two or more coexisting nutritional deficiencies. Collectively, these findings indicate that the skeletal risk profile of TGD adolescents is established prior to any hormonal intervention, with nutritional and behavioral determinants already present at the time of referral.

The lower pre-treatment BMD z-scores that had been observed in the AMAB adolescents are consistent with prior cross-sectional studies that demonstrated reduced bone mineralization in treatment-naïve AMAB youth (5–7). The median between-group difference of 0.60 z-score is clinically relevant, particularly given the evidence that GnRHa treatment may be associated with further declines in BMD z-scores during adolescence (8, 11). Sex assigned at birth remained an independent predictor of the BMD z-score after adjustment for the BMI z-score and other covariates, a finding that warrants careful interpretation. Because BMD z-scores are calculated by using sex-specific cis-gender normative reference data, the mechanism underlying this residual association is not immediately apparent. One possible explanation is that sex-related differences in skeletal characteristics, including bone geometry, cortical dimensions, and periosteal apposition, are not fully captured by areal DXA-derived BMD measurements. However, given that these parameters were not directly assessed in the present study, this interpretation remains speculative and should be evaluated in future studies incorporating volumetric and structural measures of bone strength.

Beyond the effect of sex assigned at birth, the BMI z-score emerged as the strongest independent predictor of the BMD z-score, consistent with the established role of body weight in promoting skeletal loading and bone mineral accrual during adolescence (3, 4). The numerically higher prevalence of underweight among the AMAB adolescents may contribute to their lower BMD, whereas the numerically higher prevalence of overweight and obesity among the AFAB adolescents may partly contribute to their comparatively higher BMD, possibly through greater mechanical loading, even in the context of nutritional deficiency. These findings suggest that body weight may represent one of the most clinically actionable determinants of skeletal health in treatment-naïve TGD adolescents and support routine assessment and management of undernutrition before the initiation of GnRHa therapy.

Nutritional factors represent a critical and modifiable dimension of pre-treatment skeletal risk. The median calcium intake in our study cohort was only 400 mg/d, representing 31% of the age-specific DRI, a value substantially lower than values reported in comparable TGD cohorts and in the general adolescent population (11–14), as well as less than one-half of the median intake of 899 mg/d documented in Israeli adolescents in the MABAT Youth 2015–2016 national nutrition survey (31). Whereas 24.5% of Israeli adolescents in the general population met the DRI in that survey, only 0.7% of the individuals in the present cohort had reported calcium intake reaching the DRI on the day of assessment. This highlights the very low calcium intake reported in our cohort compared with the broader Israeli adolescent population, while recognizing that a single dietary assessment cannot fully characterize habitual intake. Although reported calcium intake was not independently associated with BMD, this may reflect the limited variability in calcium intake within the cohort, as well as potential exposure misclassification related to assessment based on a single dietary recall. With nearly all individuals reporting calcium intake below the DRI on the assessment day, the ability to detect a dose-response relationship was inherently limited. The clinical relevance of this finding lies in the consistently low reported calcium intake across the cohort, as the majority of participants had intake levels below recommendations that have been linked in pediatric literature to optimal peak bone mass accrual (13–15). Collectively, these findings support the integration of nutritional assessment and calcium optimization prior to GnRHa initiation.

Vitamin D deficiency or insufficiency was similarly prevalent, affecting 91.5% of our study participants, consistent with rates reported in prior TGD cohorts (6, 9, 11). The coexistence of suboptimal vitamin D status and low reported calcium intake is of clinical relevance, as vitamin D plays a key role in calcium absorption and skeletal mineralization (13). Navabi et al. reported a direct association between baseline vitamin D status and BMD in a comparable TGD cohort (11), supporting the clinical significance of the combined deficiency observed here. Taken together, low reported calcium intake and widespread vitamin D insufficiency represent a correctable nutritional risk that should be addressed before gonadal hormone suppression is initiated. The nutritional risk profile in this study’s cohort extended beyond calcium and vitamin D. The disproportionately higher prevalence of iron deficiency in the AFAB adolescents most likely reflects ongoing menstrual blood loss prior to hormonal suppression, a clinically important and readily treatable finding that may go unrecognized without targeted screening. Borderline vitamin B12 and folate levels were also common and may reflect the lower consumption of dairy products and animal-derived foods previously described among TGD youth (16, 18), warranting monitoring given the role of these micronutrients in hematopoiesis and neurological function. The broader picture is one of systemic nutritional inadequacy. Nearly two-thirds of the study participants with complete micronutrient data had two or more co-occurring deficiencies, and fewer than 4% were unaffected across all four of the assessed markers. This pattern suggests that nutritional vulnerabilities in this population cannot be adequately addressed through single-nutrient supplementation, and reinforces the importance of comprehensive dietary assessment as a routine component of pre-treatment care.

Lifestyle factors added a further dimension to this risk profile. Physical inactivity and inadequate sleep were prevalent across the cohort and did not differ between groups, suggesting that lifestyle-related skeletal risk represents a shared vulnerability rather than a sex-specific one. The high rate of physical inactivity is not unexpected given the well-documented psychosocial barriers faced by TGD youth, including gender dysphoria in gendered sporting environments, weight-based bullying, and minority stress (16, 17). Effective promotion of physical activity in this population probably requires addressing these barriers alongside standard exercise guidance, with particular attention to gender-affirming approaches to weight-bearing activity. The below-norm MFR z-scores observed in both groups replicate the pattern of unfavorable body composition previously reported by our group in a comparable cohort from the same clinic (9). The inverse bivariate association between MFR z-score and BMD z-score most plausibly reflects the dominant positive contribution of fat mass to BMD z-score in this cohort: because MFR is calculated as muscle divided by fat mass, participants with a higher MFR had proportionally less fat and correspondingly lower BMD z-scores, a pattern supported by the positive association between fat mass percentage and BMD z-score in the same sample. This association warrants cautious interpretation and does not imply that muscle mass is detrimental to bone health. Although MFR z-score was not independently associated with BMD in multivariable analysis, this finding should be interpreted cautiously given the substantial BIA missingness (32.0%) that reduced the sample available for Model 1. Future studies incorporating more complete body composition assessment may help clarify the relative contributions of lean and fat mass to skeletal health in TGD adolescents.

### Limitations and strengths

This study has several limitations that bear mention. Its retrospective, single-center, and cross-sectional design limits generalizability and precludes causal inference. Selection bias cannot be excluded, since 37.3% of the reviewed records were excluded because of only a single clinic visit, and these individuals may represent a subgroup that is systematically different from those who continued in care. The excluded single-visit group had a significantly higher proportion of AFAB adolescents, which may represent a source of selection bias; findings should be interpreted within this context. Missing BIA and covariate data were plausibly missing at random with respect to the primary outcome, supporting the validity of the complete-case approach. In addition, BMD z-scores were derived from sex-specific reference data developed in cisgender populations. Although this approach is consistent with current clinical practice, the applicability of these normative datasets to TGD adolescents remains uncertain and may influence the interpretation of between-group comparisons. Physical activity and sleep were assessed using open-ended self-report measures rather than validated questionnaires or accelerometry, which may limit measurement precision. Psychiatric conditions and associated psychotropic medication use may influence appetite, dietary intake, body weight, physical activity, and bone health. Because detailed data on psychotropic medication exposure were not evaluated in the present analysis, their potential contribution to the observed nutritional and bone health findings cannot be excluded. The 24-h dietary recall captures a single day of intake, was conducted only on clinic weekdays without capturing weekend dietary patterns, and does not replace the gold-standard of repeated (e.g., 3-day) dietary records for estimating habitual intake; these findings may therefore not fully reflect participants’ habitual calcium consumption, although the recall was applied consistently across all participants using a validated national database. Use of vitamin D, calcium, iron, or multivitamin supplementation prior to assessment was not systematically recorded in the medical record and could not be accounted for in the nutritional and micronutrient analyses. Bone turnover markers, sex hormone levels, and grip strength values were not available, limiting mechanistic interpretation. The high rate of missing BIA data (32.0%) resulting from hardware failure reduced power for analyses incorporating the MFR z-score and may have introduced bias. BIA assessments were not performed under standardized fasting or hydration conditions, which may have introduced additional measurement variability.

The strengths of this study include the simultaneous assessment of DXA-derived BMD, validated dietary calcium intake, BIA-derived body composition, and a comprehensive micronutrient panel within a single cohort, providing an integrated characterization of skeletal and nutritional status during pre-treatment assessment. The standardized multidisciplinary assessment protocol at a single national referral center minimizes information bias and ensures consistency of measurement practices. The sample size supported both between-group comparisons and multivariable analyses, and stratification by sex assigned at birth aligns with current reporting recommendations for TGD populations.

## Conclusion

Treatment-naïve TGD adolescents present to gender clinics with multiple co-occurring and largely modifiable risk factors for impaired skeletal health, including widespread low reported calcium intake, vitamin D insufficiency, unfavorable body composition, and lifestyle-related vulnerabilities. A lower BMI appears to be a major contributor to the reduced BMD observed in TGD-Female (AMAB) adolescents, while both BMI and sex assigned at birth were independently associated with skeletal status before treatment initiation. These findings identify multiple modifiable nutritional and lifestyle factors that represent candidates for targeted pre-treatment intervention, and support the development of evidence-based pre-treatment assessment protocols for TGD adolescents. Whether such interventions improve skeletal outcomes requires prospective investigation.

## Data Availability

The datasets presented in this article are not readily available due to ethical restrictions and the need to protect participant confidentiality. Access to anonymized data may be considered upon reasonable request and subject to approval by the Institutional Review Board of Tel Aviv Sourasky University Medical Center. Requests to access the datasets should be directed to LP, liatpe@tlvmc.gov.il.
